# Recurrent Hypoglycemia in a Non-diabetic: A Case of Suspected Insulinoma Lost to Follow-Up

**DOI:** 10.7759/cureus.89291

**Published:** 2025-08-03

**Authors:** Ateetmani Pannu, Simant Shah

**Affiliations:** 1 Emergency Medicine, Inspira Medical Center Mullica Hill, Mullica Hill, USA

**Keywords:** emergency medicine resident, emergency medicine training, pancreatic insulinoma, recurrent hypoglycemia, whipple procedure

## Abstract

Insulinomas are rare insulin-secreting pancreatic neuroendocrine tumors (PNETs) that can cause profound hypoglycemia, particularly in non-diabetic patients. We report the case of a 56-year-old female who presented to the emergency department with altered mental status, a witnessed seizure, and recurrent, refractory hypoglycemia. She had no history of diabetes or hypoglycemic agent use and was reportedly in her usual state of health until the event. Initial emergency evaluation revealed persistent hypoglycemia despite dextrose administration. Cross-sectional imaging identified a pancreatic lesion concerning for insulinoma in the appropriate clinical context. Although confirmatory outpatient biochemical testing (e.g., insulin, C-peptide levels) was planned, the patient was ultimately lost to follow-up. This case underscores the critical role of emergency physicians in maintaining a broad differential when evaluating unexplained, refractory hypoglycemia, particularly when formal diagnosis is precluded.

## Introduction

Insulinomas are rare, functional pancreatic neuroendocrine tumors (PNETs) that originate from beta cells of the islets of Langerhans and are characterized by unregulated insulin secretion, leading to recurrent episodes of hypoglycemia. These tumors are typically small, solitary, and benign in over 90% of cases, but their clinical presentation can be elusive and variable. The estimated incidence of insulinoma is approximately 1-4 cases per million people annually, accounting for 1-2% of all pancreatic neoplasms [1].

The hallmark symptoms of insulinomas result from neuroglycopenia - seizures, visual disturbances, behavioral changes, and even loss of consciousness - making them easily mistaken for primary neurologic or psychiatric conditions [2]. If left unrecognized, repeated episodes of hypoglycemia can result in permanent neurologic injury and significant morbidity [3]. Prompt diagnosis and intervention are critical in the emergency setting, where delayed recognition of atypical hypoglycemia may lead to irreversible neurologic sequelae [3, 4].

This case explores the emergency department evaluation of a non-diabetic patient with recurrent, unexplained hypoglycemia and a clinical picture concerning for insulinoma, underscoring the importance of early clinical suspicion even when the definitive diagnosis is not immediately attainable.

## Case presentation

A 56-year-old female with a history of hypertension presented to the emergency department (ED) after experiencing a witnessed seizure while traveling. According to family members, she had multiple episodes of altered consciousness without return to baseline mentation, prompting emergency medical services (EMS) activation. Upon EMS arrival, the patient was obtunded, with a point-of-care glucose of 18 mg/dL. She received 250 mL of dextrose 10% (D10), resulting in improvement of both her mental status and serum glucose.

Upon ED arrival, the patient again became diaphoretic and confused, with a repeat glucose of 14 mg/dL. A second D10 bolus was administered with resolution of symptoms. She denied any history of diabetes, seizure disorders, recent medication changes, supplement use, alcohol intake, or illicit drug use. She did report eliminating sugar from her diet one month earlier due to pre-diabetes concerns. A review of systems was negative for chest pain, gastrointestinal symptoms, or recent illnesses.

Vital signs were notable for mild hypothermia (34.3°C) and tachypnea (respiratory rate of 23). Physical examination, including a complete neurologic exam, was non-focal. Despite the patient’s return to baseline mentation, the witnessed seizure and prior unresponsiveness warranted further evaluation for alternative causes of her altered mental status, including structural central nervous system (CNS) pathology. A non-contrast computed tomography (CT) scan of the brain was obtained to evaluate for possible acute intracranial abnormalities (e.g., seizure focus, mass lesion, or infarction), and was unremarkable.

Given the patient’s recurrent hypoglycemia in the setting of abnormal vital signs, additional laboratory workup was pursued to assess systemic causes such as infection, ischemia, or metabolic derangements. A complete blood count revealed leukocytosis with monocyte predominance, which, although nonspecific, raised consideration for infectious triggers of hypoglycemia (Table 1). However, infectious workup - including a urinalysis and a comprehensive respiratory viral panel - was unremarkable (Tables 2-3). A complete metabolic panel was also obtained to evaluate for electrolyte abnormalities (e.g., hyponatremia-induced seizure) and was within normal limits (Table 4). Despite the absence of chest pain or anticoagulation use, troponin and coagulation studies were obtained due to her altered mental status and abnormal vital signs - hypothermia and tachypnea - both of which may indicate systemic illness, infarction, or sepsis; these studies were also unremarkable (Tables 5-6).

**Table 1:** Complete Blood Count HGB: hemoglobin; HCT: hematocrit; MCV: mean corpuscular volume; MCH: mean corpuscular hemoglobin; MCHC: mean corpuscular hemoglobin concentration; RDW: red cell distribution width; MPV: mean platelet volume

	Patient’s Value	Normal Range	Abnormal Values
WBC	16 (cells/µL)	4.0 - 11.0 (cells/µL)	16 (cells/µL)
RBC	4.41 (million cells/µL)	3.50 - 5.10 (million cells/µL)	
HGB	13.3 (g/dL)	11.0 - 15.2 (g/dL)	
HCT	41 (%)	32.0 - 45.0 (%)	
MCV	93 (fL)	80 - 98.0 (fL)	
MCH	30.2 (pg/cell)	27.6 - 34.5 (pg/cell)	
MCHC	32.4 (g/dL)	33.0 - 36.0 (g/dL)	
RDW	12 (fL)	11.0 - 15.0 (fL)	
Platelets	348 (µL)	140 - 380 (µL)	
MPV	10 (fL)	9.4 - 12.4 (fL)	
Neutrophil Auto	68.4 (cells/µL)	40.0 - 74.0 (cells/µL)	
Lymphocyte Auto	19.1 (cells/µL)	19.0 - 48.0 (cells/µL)	
Monocyte Auto	11.3 (cells/µL)	3.0 - 9.0 (cells/µL)	11.3 (cells/µL)
Eosinophil Auto	0.2 (cells/µL)	0.0 - 7.0 (cells/µL)	
Basophil Auto	0.5 (cells/µL)	0.0 - 1.0 (cells/µL)	
IG Auto	0.5 (cells/µL)	0.0 - 1.0 (cells/µL)	
NRBC Auto	0 (µL)	0.0 - 0.0 (µL)	
Neutrophil Absolute	11 (cells/µL)	1.8 - 7.0 (cells/µL)	11 (cells/µL)
Lymphocyte Absolute	3.1 (cells/µL)	1.0 - 4.8 (cells/µL)	
Monocyte Absolute	0.8 (cells/µL)	0.0 - 0.8 (cells/µL)	
Eosinophil Absolute	0.03 (cells/µL)	0.0 - 0.45 (cells/µL)	
Basophil Absolute	0.1 (µL)	0.0 - 0.2 (µL)	
IG Absolute	0.08 (g/dL)	0.0 - 0.03 (g/dL)	0.08 (g/dL)

**Table 2:** Urine Analysis and Urine Drug Screen U: urine; UA: urinalysis; PCP: phencyclidine

	Patient’s Value	Normal Range
U Amphetamine screen	Negative	Negative
U Barbiturate screen	Negative	Negative
U Benzodiazepine	Negative	Negative
U Cannabis screen	Negative	Negative
U Cocaine screen	Negative	Negative
U Methadone	Negative	Negative
U Opiate screen	Negative	Negative
U PCP screen	Negative	Negative
Urine analysis		
UA Color	Yellow	Yellow
UA Appearance	Clear	Clear
UA Glucose	Negative	Negative
UA Bilirubin	Negative	Negative
UA Ketones	Negative	Negative
UA Specific Gravity	1.013	1.001 - 1.030
UA Blood	Negative	Negative
UA pH	6	5.0 - 8.0
UA Protein	Negative	Negative
UA Urobilinogen	0.2	0.2 - 1.0
UA Nitrite	Negative	Negative
UA Leukocyte Esterase	Trace	Negative

**Table 3:** Respiratory Viral Panel

	Patient’s Value	Normal Range
SARS-CoV-2 (COVID-19)	Negative	Negative
MRSA DNA probe	Negative	Negative
Adenovirus	Negative	Negative
Coronavirus 229E	Negative	Negative
Coronavirus HKU1	Negative	Negative
Coronavirus NL63	Negative	Negative
Coronavirus OC43	Negative	Negative
Metapneumovirus	Negative	Negative
Influenza A	Negative	Negative
Influenza B	Negative	Negative
Parainfluenza virus 1	Negative	Negative
Parainfluenza virus 2	Negative	Negative
Parainfluenza virus 3	Negative	Negative
Parainfluenza virus 4	Negative	Negative
Respiratory Syncytial Virus A+B	Negative	Negative
Rhinovirus/Enterovirus	Negative	Negative
*Bordetella parapertussis*	Negative	Negative
*Bordetella pertussis*	Negative	Negative
*Chlamydophila pneumoniae*	Negative	Negative
*Mycoplasma pneumoniae*	Negative	Negative

**Table 4:** Complete Metabolic Panel BUN: blood urea nitrogen

	Patient’s Value	Normal Range
Glucose Level	88 (mmol/L)	74 - 106 (mmol/L)
BUN	20 (mg/dL)	9.0 - 23 (mg/dL)
Creatine Level	0.67 (mg/dL)	0.55 - 1.02 (mg/dL)
BUN/Creatinine Ratio	29.9 (mg/dL)	5.0 - 35.0 (mg/dL)
Sodium Level	140 (mEq/L)	136 - 145 (mEq/L)
Potassium Level	3.7 (mEq/L)	3.4 - 4.5 (mEq/L)
Chloride Level	108 (mmol/L)	98 - 107 (mmol/L)
CO_2_	22.9 (mEq/L)	22.0 - 30.0 (mEq/L)
Anion Gap	9 (mEq/L)	6.0 - 16.0 (mEq/L)
Osmolality Calculation	281 (mOsm/kg)	275 - 295 (mOsm/kg)
Calcium Level	8.8 (mmol/L)	8.4 - 10.2 (mmol/L)
Protein Total	6.9 (g/dL)	5.7 - 8.2 (g/dL)
Albumin Level	4.4 (g/dL)	3.5 - 5.0 (g/dL)
Globulin	2.5 (g/dL)	3.5 - 5.0 (g/dL)
A/G Ratio	1.8	0.8 - 2.0
Alk Phos	92 (IU/L)	46 - 116 (IU/L)
ALT	27 (U/L)	10.0 - 49.0 (U/L)
AST	30 (U/L)	0 - 34 (U/L)
Bilirubin Total	0.2 (mg/dL)	0.2 - 1.3 (mg/dL)
Bilirubin Direct	0.1 (mg/dL)	0.0 - 0.3 (mg/dL)

**Table 5:** Troponin Enzymes HS: high sensitivity

	Patient’s Value	Normal Range
HS Troponin Baseline	< 3 (ng/L)	< 3 (ng/L)
HS Troponin 2 Hours	6 (ng/L)	< 3 (ng/L)
Delta Baseline 2 Hours	No Calc	< 3 (ng/L)

**Table 6:** Coagulation Studies PT: prothrombin time; INR: international normalized ratio; PTT: partial thromboplastin time

	Patient’s Value	Normal Range
PT	9.8 (s)	9.7 - 13.3 (s)
INR	0.9	0.8 - 1.2
PTT	27.3 (s)	22.2 - 37.6 (s)

**Figure 1:**
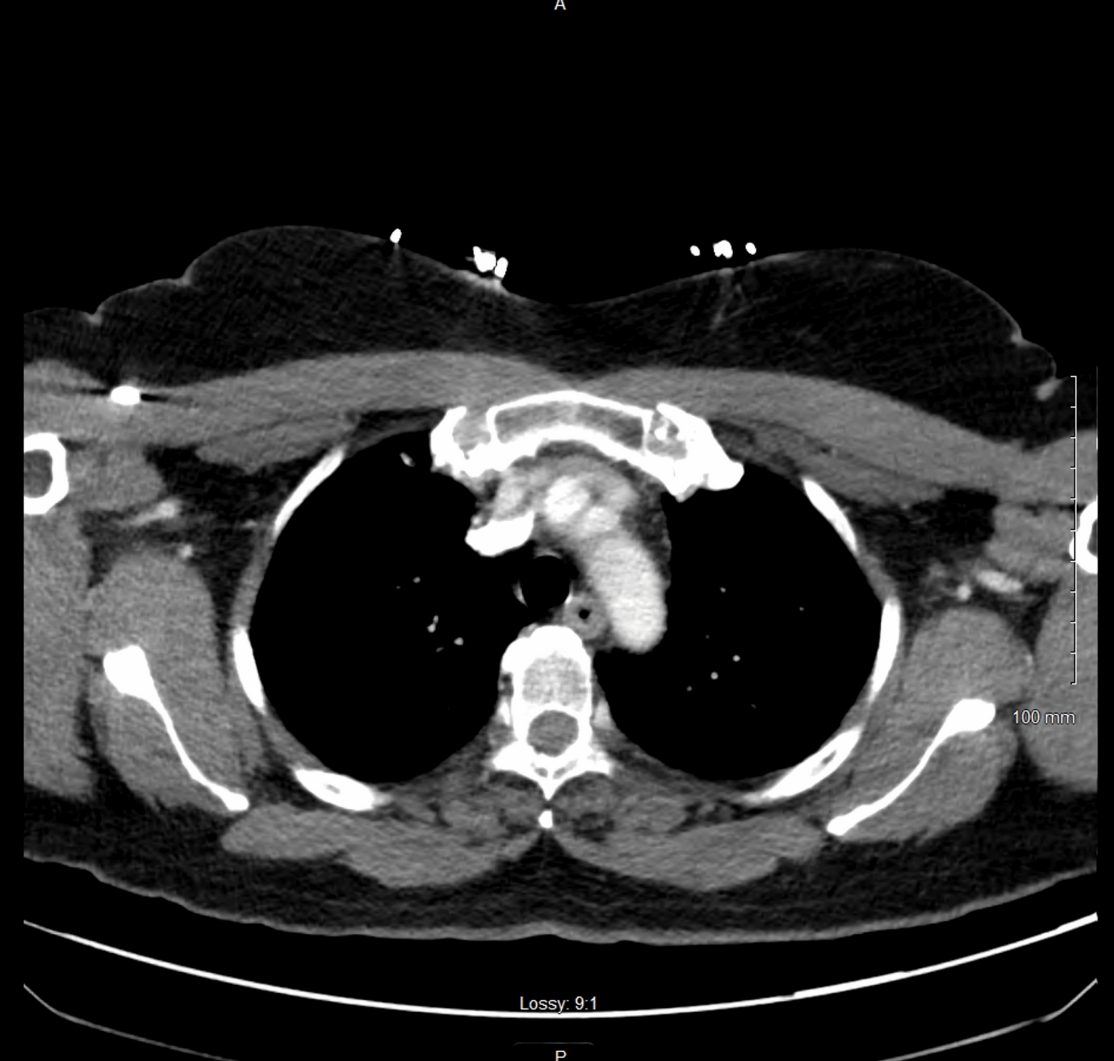
Computed Tomography of Abdomen and Pelvis

Following the second D10 bolus, the patient’s repeat serum glucose was 88 mg/dL. However, given the refractory nature of her hypoglycemia and absence of identifiable iatrogenic or toxicologic causes, a CT of the abdomen and pelvis was performed to evaluate for potential endocrine, hepatic, or malignant pathology. Imaging revealed a 1.2-cm cystic lesion at the pancreatic body-tail junction without associated ductal dilation (Figure 1). Chest X-ray and CT thorax were negative for acute findings.

The patient was admitted to the intensive care unit (ICU) on a dextrose 5% in water (D5W) infusion for persistent glucose instability. During her ICU stay, the patient disclosed prior semaglutide (Ozempic) use, which she had discontinued one month earlier due to gastrointestinal side effects. Although large-scale clinical trials and real-world observational studies have not demonstrated a definitive association between semaglutide use and pancreatic neoplasia, preclinical models suggest a theoretical risk of glucagon-like peptide-1 (GLP-1) receptor agonists contributing to the proliferation of neuroendocrine tissue - particularly in lesions expressing GLP-1 receptors. This consideration prompted heightened clinical vigilance during evaluation of her pancreatic lesion. [5, 6].

Given the patient’s recurrent hypoglycemia in the absence of diabetes or hypoglycemic agents and the pancreatic lesion on imaging, insulinoma was strongly suspected. Outpatient biochemical testing, including serum insulin, C-peptide, proinsulin, and sulfonylurea screen, was planned to confirm the diagnosis. Unfortunately, the patient was lost to follow-up before a confirmatory workup or surgical evaluation could be completed.

## Discussion

Insulinomas are the most common functioning PNETs, characterized by inappropriate and autonomous insulin secretion, often resulting in symptoms of neuroglycopenia such as confusion, seizure, and altered mentation. In non-diabetic individuals, unexplained hypoglycemia - particularly when symptoms improve with glucose administration - should raise strong clinical suspicion for insulinoma, as outlined by Whipple’s Triad [2].

This case highlights the diagnostic challenges insulinomas can pose in the emergency setting, especially when patients present with altered mental status or seizure activity in the absence of common risk factors. Our patient had no history of diabetes, seizure disorders, oral hypoglycemic agents, insulin use, or critical illness to explain her profound hypoglycemia. The repeated cycle of symptomatic resolution followed by recurrent hypoglycemia despite dextrose administration and the presence of a cystic pancreatic lesion on imaging heightened concern for an insulin-secreting tumor.

While laboratory testing for insulin, C-peptide, and sulfonylurea levels was not completed in the ED, the clinical scenario strongly suggested endogenous hyperinsulinemic hypoglycemia. In the absence of exogenous insulin or sulfonylurea use, and with no evidence of adrenal insufficiency or liver dysfunction, insulinoma remained the leading differential diagnosis. Although the patient met systemic inflammatory response syndrome (SIRS) criteria - including hypothermia, tachypnea, and leukocytosis - all infectious studies, including urinalysis, chest imaging, and a comprehensive respiratory viral panel, were negative. Blood cultures were not drawn in the ED, which is a limitation; however, the patient demonstrated rapid clinical improvement with glucose administration alone, without antibiotic therapy, making sepsis a less likely etiology.

Cross-sectional imaging, particularly CT of the abdomen and pelvis, was instrumental in narrowing the differential diagnosis. While endoscopic ultrasound or magnetic resonance imaging (MRI) are often used to further characterize pancreatic lesions, CT remains the preferred initial modality in the ED due to its accessibility and high detection rates for insulinomas [7].

Early recognition of insulinoma in the emergency setting is crucial, particularly given the nonspecific and neurologically mimicking nature of its presentation. Emergency physicians may be the first to detect subtle red flags and initiate appropriate diagnostic pathways, even when confirmatory biochemical testing is deferred to the outpatient setting [3].

Although our patient was lost to follow-up, this case underscores the importance of initiating the workup early. Non-metastatic insulinomas, once resected, typically have an excellent prognosis with survival rates reaching the general population. However, some studies suggest an increased risk of subsequent comorbidities - including atrial fibrillation, intestinal obstruction, and possibly breast and renal cancers - among patients with PNETs [8]. While causality remains unconfirmed, these associations may reflect broader neuroendocrine dysregulation and merit consideration in long-term surveillance strategies. For this patient, earlier definitive diagnosis and close follow-up could have led to timely intervention and improved long-term care planning.

## Conclusions

Recurrent hypoglycemia in non-diabetic patients warrants consideration of insulinoma, particularly when no exogenous or iatrogenic cause is identified. Emergency physicians should be familiar with Whipple’s Triad and the initial workup for endogenous hyperinsulinemia. Early recognition and stabilization in the ED are critical, given the potential for neurologic injury and diagnostic delays. This case reinforces the importance of diagnostic vigilance and maintaining a broad differential when evaluating unexplained hypoglycemia while also highlighting the essential role of emergency clinicians in identifying rare but serious conditions before they become life-threatening.
